# Endothelial CCR6 expression due to FLI1 deficiency contributes to vasculopathy associated with systemic sclerosis

**DOI:** 10.1186/s13075-021-02667-9

**Published:** 2021-11-13

**Authors:** Tetsuya Ikawa, Takuya Miyagawa, Yuki Fukui, Satoshi Toyama, Jun Omatsu, Kentaro Awaji, Yuta Norimatsu, Yusuke Watanabe, Ayumi Yoshizaki, Shinichi Sato, Yoshihide Asano

**Affiliations:** Department of Dermatology, University of Tokyo Graduate School of Medicine, Tokyo, Japan

**Keywords:** Systemic sclerosis, CCL20, CCR6, FLI1, Endothelial cells

## Abstract

**Background:**

We have recently demonstrated that serum CCL20 levels positively correlate with mean pulmonary arterial pressure in patients with systemic sclerosis (SSc). Considering a proangiogenic effect of CCL20 on endothelial cells via CCR6, the CCL20/CCR6 axis may contribute to the development of SSc vasculopathy. Therefore, we explored this hypothesis using clinical samples, cultured cells, and murine SSc models.

**Methods:**

The expression levels of CCL20 and CCR6 in the skin, mRNA levels of target genes, and the binding of transcription factor FLI1 to the target gene promoter were evaluated by immunostaining, quantitative reverse transcription PCR, and chromatin immunoprecipitation, respectively. Vascular permeability was evaluated by Evans blue dye injection in bleomycin-treated mice. Angiogenic activity of endothelial cells was assessed by in vitro angiogenesis assay.

**Results:**

CCL20 expression was significantly elevated in dermal fibroblasts of patients with early diffuse cutaneous SSc, while CCR6 was significantly up-regulated in dermal small vessels of SSc patients irrespective of disease subtypes and disease duration. In human dermal microvascular endothelial cells, *FLI1* siRNA induced the expression of CCR6, but not CCL20, and FLI1 bound to the *CCR6* promoter. Importantly, vascular permeability, a representative SSc-like vascular feature of bleomycin-treated mice, was attenuated by *Ccr6* siRNA treatment, and *CCR6* siRNA suppressed the angiogenic activity of human dermal microvascular endothelial cells assayed by in vitro tube formation.

**Conclusions:**

The increased expression of endothelial CCR6 due to FLI1 deficiency may contribute to the development of SSc vasculopathy.

## Background

Systemic sclerosis (SSc) is a multisystem autoimmune disease representing vasculopathy and tissue fibrosis of the skin and various internal organs [1]. Recent clinical studies have demonstrated the efficacy of several drugs for tissue fibrosis and vasculopathy associated with SSc. For instance, tocilizumab and bosentan prevent the decrease in the percentage of vital capacity and the onset of new digital ulcers, respectively [2, 3]. Also, several new therapeutic candidates are now under the clinical trials [4], but the identification of new therapeutic targets is quite important to further facilitate the development of new therapies against SSc.

Chemokines have attracted much attention as a potential therapeutic target family of molecules in the field of SSc based on the results of clinical studies and animal models [5]. With respect to CCL20, we have recently found that serum CCL20 levels correlate with mean pulmonary arterial pressure (mPAP) in SSc patients [6], suggesting that the CCL20/CCR6 axis underlies the developmental mechanism of SSc vasculopathy. According to previous studies, the CCL20/CCR6 axis promotes the chemotaxis of immature dendritic cells, T helper (Th) 17 cells, regulatory T cells, and B cells under both homeostatic and inflammatory conditions [7], contributing to the maintenance of homeostatic immune balance and the development of pathologic inflammation, such as psoriasis [8, 9], atopic dermatitis [9], inflammatory bowel disease [10], systemic lupus erythematosus [11], dermatomyositis/polymyositis [12], and SSc [13]. On the other hand, an accumulating body of evidence indicates that the CCL20/CCR6 axis regulates endothelial behaviors related to tumor angiogenesis. For instance, Hippe et al. revealed the following findings: (i) CCL20 expression levels in tumors correlate with advanced tumor stage, increased lymph node metastasis, and decreased survival; (ii) microvascular endothelial cells abundantly express CCR6; (iii) CCR6 signaling in endothelial cells induces angiogenesis; and (iv) tumor growth and tumor-associated vascularization are decreased in CCR6-deficient mice due to its deficiency in stromal cells, but not within the immune system [14]. Thus, the CCL20/CCR6 axis is involved in the development of inflammatory diseases and tumor angiogenesis, but its role remains unknown in vascular disorders, including SSc vasculopathy.

Friend leukemia virus integration 1 (FLI1) is a member of the ETS transcription factor family, the expression of which is broadly suppressed in various cell types in SSc lesional and non-lesional skin [15, 16]. Since FLI1 expression is genetically and epigenetically suppressed in SSc patients [17, 18], FLI1 deficiency likely serves as a predisposing factor in SSc development. Supporting this notion, FLI1 deficiency induces SSc-like phenotypes in dermal fibroblasts, endothelial cells, macrophages, keratinocytes, and dermal dendritic cells [16, 19–25]. Therefore, the molecular analysis based on FLI1 deficiency provides us with a useful clue to know the significance of target molecules in the development of SSc. Indeed, this research strategy has been already applied to the study of chemokines in SSc. For instance, FLI1 deficiency induces CXCL5 upregulation and CXCL6 downregulation in endothelial cells [26, 27]. In addition, CXCL13 expression is enhanced by FLI1 deficiency in macrophages [24]. Importantly, the analyses on serum levels of these chemokines suggest their contribution to the development of tissue fibrosis, vasculopathy, and/or immune activation in SSc. On the other hand, FLI1 deficiency is also implicated in the development of pulmonary arterial hypertension (PAH) by modulating the expression of angiogenesis-related genes [28]. Taken together with our recent data regarding the association of the CCL20/CCR6 axis with SSc-PAH [6], we hypothesized that FLI1 deficiency regulates the CCL20/CCR6 axis in the context of SSc vasculopathy.

Based on these backgrounds, we investigated the potential role of CCL20/CCR6 in SSc vasculopathy and the contribution of FLI1 deficiency to this process by a series of experiments with clinical samples, cultured endothelial cells, and animal models.

## Methods

### Immunohistochemistry

Immunohistochemistry was performed on formalin-fixed, paraffin-embedded skin sections with Vectastain Elite ABC kit (Vector Laboratories, Burlingame, CA, USA) and antibodies against CCL20 (Thermo Fisher Scientific, Waltham, MA, USA), CCR6 (Abcam, Cambridge, UK), α-smooth muscle actin (α-SMA) (Abcam), NG2 (Abcam), vascular endothelial (VE)-cadherin (Thermo Fisher Scientific), and cleaved caspase 3 (Cell Signaling Technology, Danvers, MA, USA). Antigen retrieval was performed using Dako Target Retrieval Solution pH 9 (Dako North America, Inc., Carpinteria, CA, USA). Skin samples were obtained from forearms of 6 diffuse cutaneous SSc (dcSSc) patients, 6 limited cutaneous SSc (lcSSc) patients, and 5 healthy controls. Patients’ demographic features are shown in Table 1. All patients with dcSSc were the early subtype (disease duration of < 2 years). Horseradish peroxidase activity was detected by 3, 3′-diaminobenzidine. Counterstaining was conducted with methyl green. Manual scoring of staining intensity was graded as follows: −, no staining; +, slight staining; ++, moderate staining; +++, strong staining. Capillaries and venules were determined based on their diameter. Blood vessels with diameter almost equal to or less than that of erythrocyte were classified as capillaries, and the others were classified as venules [29]. The manual scoring of staining intensity and the determination of blood vessel type were performed by two independent dermatologists in a blinded manner (T. Ikawa and Y. Asano).

**Table 1:** Expression profiles of CCL20 and CCR6 in skin sections of SSc patients and healthy controls

						CCL20				CCR6			
Samples		Age/sex	Disease duration (year)	Skin score (mRSS)	Autoantibodies	Keratinocytes	Fibroblasts	Blood vessels	Infiltrated cells	Keratinocytes	Fibroblasts	Blood vessels	Infiltrated cells
HC	1	38/F				++	–	++	+	–	–	+	+
	2	25/F				+	–	++	++	+	–	+	++
	3	21/M				+	–	++	++	++	–	++	++
	4	22/M				++	+	++	++	–	–	–	–
	5	30/F				+	++	++	++	++	+	+	+
SSc	dcSSc1	76/M	0.5	7	Topo-I	+	+++	+++	++	++	–	+	+
	dcSSc2	44/F	0.3	12	Topo-I	+	+	++	++	++	–	+	+
	dcSSc3	40/M	0.5	31	Topo-I	++	+++	++	++	++	++	++	++
	dcSSc4	59/F	0.3	7	Topo-I	++	+++	+++	+++	+++	–	++	++
	dcSSc5	74/F	1	15	U1RNP	++	+++	++	++	++	–	++	++
	dcSSc6	35/F	1.5	12	Topo-I	+	–	+	+	++	–	++	+
	lcSSc1	47/F	5	2	Topo-I	++	++	++	++	+	–	++	++
	lcSSc2	32/F	0.2	6	Topo-I, U1RNP	++	+	++	++	++	++	+++	+++
	lcSSc3	45/F	6	6	ACA	++	–	++	++	++	–	++	+
	lcSSc4	75/F	25	6	ACA	++	+	++	++	++	–	++	++
	lcSSc5	57/F	2	2	ACA	++	+++	++	++	+++	–	+++	+++
	lcSSc6	81/F	4	2	ACA	++	–	++	++	+++	++	+++	+++

We used the following grading system for the immunohistochemistry: −, no staining; +, slight staining; ++, moderate staining; +++, strong staining. *HC* healthy control; *lcSSc* limited cutaneous systemic sclerosis; *dcSSc* diffuse cutaneous systemic sclerosis; *mRSS* modified Rodnan total skin thickness score; *Topo-I* anti-topoisomerase I antibody; *U1RNP* anti-U1RNP antibody; *ACA* anticentromere antibody

### Gene silencing of *FLI1*

Human dermal microvascular endothelial cells (HDMECs) (Lonza, Walkersville, MD, USA) were cultured on collagen-coated tissue culture plates in Endothelial Basal Medium-2 supplemented with the Endothelial Cell Growth Medium-2 Bullet Kit (Lonza). Shortly after seeded, HDMECs were transfected with *FLI1* siRNA or non-silencing scrambled RNA (SCR) (10 nM, both purchased from Santa Cruz Biotechnology, Santa Cruz, CA, USA) mixed with HiPerFect Transfection Reagent (Qiagen, Valencia, CA, USA) for 48 h. Some cells were stimulated with recombinant human IL-17A, IL-10, and TGF-β1 (all from Peprotech, Rocky Hill, NJ, USA). Cells were then collected using TRIzol Reagent (Thermo Fisher Scientific) for RNA isolation.

### RNA isolation and quantitative reverse transcription (qRT)-PCR

RNA isolation from HDMECs and qRT-PCR were conducted as described previously [30]. The sequences of primers were as follows: *FLI1*-forward 5′-GGATGGCAAGGAACTGTGTAA-3′, *FLI1*-reverse 5′-GGTTGTATAGGCCAGCAG-3′, *CCL20*-forward 5′- TTGTGCGTCTCCTCAGTAAAAA-3′, *CCL20*-reverse 5′- GCAAGTGAAACCTCCAACCC-3′; *CCR6*-forward 5′- GGGGGCTGTCAGTCATCATC-3′, *CCR6*-reverse 5′- CGTAGAGCACAGGGTTCAGG-3′; *GAPDH*-forward 5′-ACCCACTCCTCCACCTTTGA-3′, *GAPDH*-reverse 5′-CATACCAGGAAATGAGCTTGACAA-3′.

### Immunoblotting

Whole cell lysates of HDMECs treated with *FLI1* siRNA or SCR were subjected to sodium dodecyl sulfate-PAGE (Thermo Fisher Scientific) and immunoblotting. Antibodies used were against β-actin (Santa Cruz Biotechnology), CCR6, and FLI1 (both from Abcam), followed by horseradish peroxidase (HRP)-conjugated secondary antibody (Cell Signaling Technology). The protein bands were visualized with chemiluminescence (Nacalai Tesque, Kyoto, Japan). The density of each band was quantified with ImageJ software (National Institutes of Health).

### Chromatin immunoprecipitation (ChIP) assay

ChIP assay was conducted using EpiQuik ChIP kit (Epigentek, Farmingdale, NY, USA) as described previously [30]. Putative FLI1 binding site in the *CCR6* promoter was predicted using a web site, JASPAR. The primers that amplify fragments of the *CCR6* transcript variant 1 (− 252 bp to − 39 bp) and transcript variant 2 (− 1395 bp to − 1186 bp) were as follows: *CCR6* transcript variant 1/Forward, 5′- ACTGCCGTATCCCTTGTGC-3′; *CCR6* transcript variant 1/Reverse, 5′- TGGGAGAATGGACATTGTGACC-3′, *CCR6* transcript variant 2/Forward, 5′- TTCTTTCCAGGCAGGCATTG-3′; *CCR6* transcript variant 2/Reverse, 5′- TCCTCCTCATTTCTACCATCGC-3′. The amplified DNA products were resolved by agarose gel electrophoresis.

### In vivo local gene silencing of *Ccr6* with atelocollagen

*Ccr6* siRNA was transfected to murine skin in vivo using atelocollagen (AteloGene® Local Use “Quick Gelation,” Koken, Tokyo, Japan). Ten micromolar of Silencer select *Ccr6* siRNA or SCR (both from Thermo Fisher Scientific) was mixed with atelocollagen, 150 μL of which was subcutaneously given to shaved lower back of wild-type (WT) mice once a week. After the injection of siRNA, 200 mg of BLM was subcutaneously injected to the same place every day for a week or 4 weeks.

### In vivo vascular permeability assay

Evans blue dye (0.5%) (Sigma-Aldrich, St. Louis, MO, USA) in 200 μl of 0.9% saline was injected into the tail vein, and mice were sacrificed in half an hour. The presence of vascular leakage was macroscopically evaluated in the skin.

### In vitro transwell permeability assay

Twenty-four well dishes with inserts (collagen-coated, 0.45-mm-meshed) were used for the assay. HDMECs (3 × 10^5^ in 300 mL of endothelial growth medium) were seeded into each insert. Cells were cultured at 37 °C for 48 h to make a monolayer of the cells at the bottom. Then, *CCR6* siRNA (final concentration, 10 nM) was added to the top chamber and cultured for another 48 h. Next, cells were stimulated with 100 ng/mL of recombinant human tumor necrosis factor-α (R&D systems, Minneapolis, MN, USA) for 24 h. After the stimulation, the bottom chambers were refilled with 1 mL of serum-free media, while serum-free media including 15 ml/mL of Streptavidin-HRP (R&D systems) were added in the top chambers. After 30-min incubation at 37 °C, 20 mL of media from the bottom chambers were transferred to a new 96-well plate in triplicate. Then, 50 mL of TMB substrate (Abcam) was added in each well and incubated for 5 min at room temperature. The reaction was stopped by adding 25 ml of stop solution (Abcam) into each well, and absorption at 450 nm was measured.

### In vitro angiogenesis assay

HDMECs were treated with 20 nM of *CCR6* siRNA or SCR (both from Thermo Fisher Scientific) for 48 h. Then, cells were treated with 10 μg/mL of mitomycin C (Sigma Aldrich) for 2 h. A 24-well plate was coated with 250 μL of growth factor-reduced Matrigel (BD Biosciences, San Jose, CA, USA). After the gel was solidified, cells were trypsinized and seeded onto the Matrigel at 7 × 10^4^ cells per well and incubated for 24 h. Cells were treated with calcein AM before observation. Five photographs were taken randomly from each well. The numbers of meshes, tubes, and intersections were counted manually.

### Statistical analysis

Statistical analysis was conducted with Welch’s *t* test to compare two unpaired data. Statistical significance was defined as a *P* value of < 0.05.

## Results

### The expression profiles of CCL20 and CCR6 in the involved skin of SSc patients

**Fig. 1:**
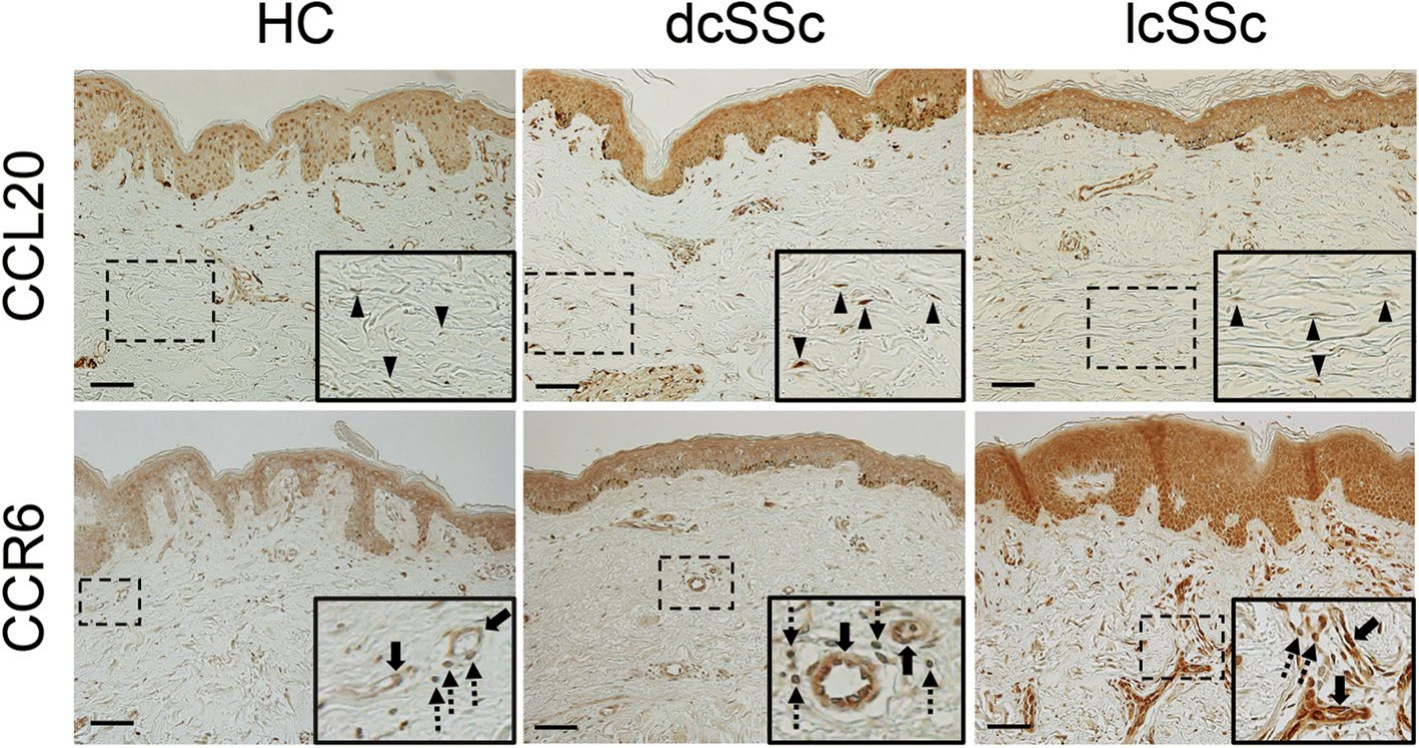
The expression profiles of CCL20 and CCR6 in SSc patients and healthy controls. The expression of CCL20 and CCR6 was evaluated by immunohistochemistry in the skin sections from diffuse cutaneous SSc (dcSSc) patients, limited cutaneous SSc (lcSSc) patients, and healthy controls (HC). Representative results are shown. Lower panels of each picture exhibit dermal fibroblasts (pointed out with arrowheads) in CCL20 staining and dermal small vessels and inflammatory cells (pointed out with unbroken arrows and dashed arrows, respectively) in CCR6 staining, which are shown with dashed solid squares in upper panels. Horseradish peroxidase activity was detected by 3, 3′-diaminobenzidine. Counterstaining was carried out with methyl green. A scale bar is 100 μm

As an initial experiment, we evaluated the expression of CCL20 and CCR6 in skin biopsy samples of SSc patients and healthy controls (Fig. 1 and Table 1). In healthy control skin CCL20 was abundantly expressed in keratinocytes, dermal small vessels, and inflammatory cells, while marginally detected in dermal fibroblasts, as previously reported [31]. In the skin of SSc patients, similar expression profiles to those of healthy controls were observed in keratinocytes, dermal small vessels and inflammatory cells, but an increased trend of CCL20 expression was evident in dermal fibroblasts relative to those cells of healthy control skin (the evaluation with grading scale; median [25–75 percentiles], 1.5 [0.25–3.0] versus 0 [0–1.5], *p* = 0.078). Importantly, CCL20 was significantly up-regulated in dermal fibroblasts of dcSSc patients (disease duration of < 2 years) compared with those cells of healthy controls (3 [0.75–3] versus 0 [0–1.5], *p* = 0.046), which is consistent with a previous finding that CCL20 expression is increased in dermal fibroblasts of early SSc patients [13]. With respect to CCR6, there were detectable signals in various cell types of healthy control skin, but dermal fibroblasts displayed a low expression level relative to the other cell types. In the skin of SSc patients, CCR6 expression was significantly increased in dermal small vessels and keratinocytes as compared to those cells of healthy control skin (2 [2–2.8] versus 1 [0.5–1.5], *p* = 0.022; 2.0 [2.0–3.0] versus 1.0 [1.0–2.0], *p* = 0.0089; respectively), while comparable in dermal fibroblasts and inflammatory cells. We also explored the association between the signal intensity of CCL20/CCR6 in each cell type and modified Rodnan total skin thickness score, but there were no significant correlations (data not shown). Taken together, these results suggest the potential contribution of dermal fibroblasts of early SSc patients to recruiting Th17 cells to the affected skin lesion, as previously reported [13], and the activation of CCL20/CCR6 axis in endothelial cells and keratinocytes of SSc-involved skin. The role of the CCL20/CCR6 axis has been well studied in endothelial cells, while the CCL20/CCR6 axis does not affect the migration and proliferation of keratinocytes [32]; therefore, we focused on endothelial cells in the following experiments.

### FLI1 deficiency induces CCR6 expression in dermal microvascular endothelial cells

**Fig. 2:**
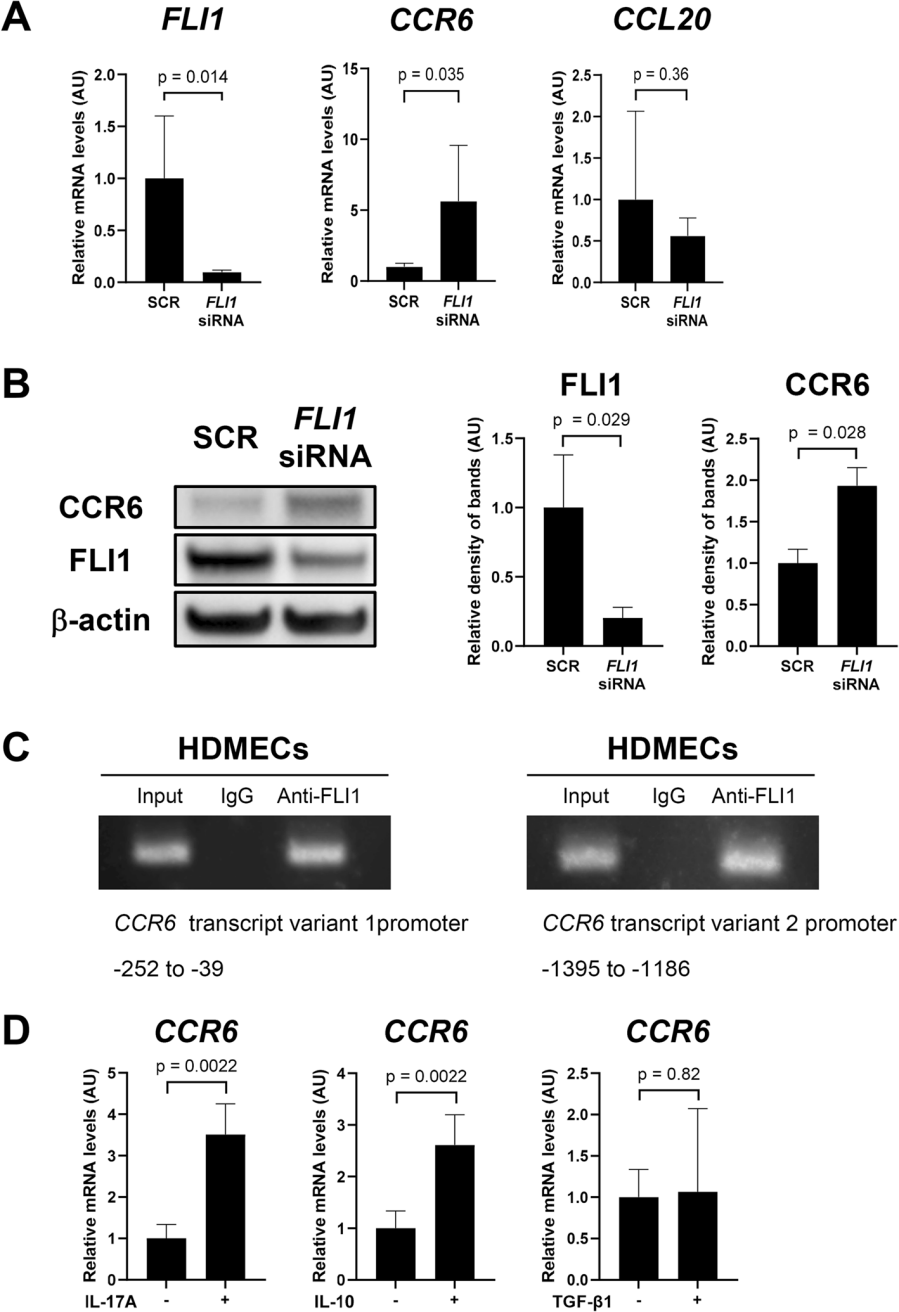
The contribution of Fli1 deficiency to the up-regulated expression of CCR6 in endothelial cells. **A** mRNA levels of *FLI1*, *CCL20*, and *CCR6* in human dermal microvascular endothelial cells (HDMECs) transfected with *FLI1* siRNA or non-silencing scrambled RNA (SCR) were examined by qRT-PCR (*n* = 6 for each group). Results are expressed as means ± SEM. AU, arbitrary unit. **B** Protein levels of CCR6 and FLI1 in HDMECs transfected with *FLI1* siRNA or SCR were examined by immunoblotting (*n* = 4 for each group). **C** Chromatin was isolated from HDMECs and immunoprecipitated using rabbit anti-FLI1 antibody or rabbit IgG. After isolation of bound DNA, PCR amplification was carried out using two sets of primers specific for the promotes of CCR6 transcript variant 1 (NM_004367) and CCR6 transcript variant 2 (NM_031409). **D** mRNA levels of CCR6 were determined by qRT-PCR in HDMECs treated with IL-17A (20 ng/mL), IL-10 (20 ng/mL), or TGF-β1 (2 ng/mL)

Given that Fli1 deficiency reproduces SSc-like properties, including the expression profiles of chemokines, in endothelial cells [26, 27], we examined the effect of *FLI1* siRNA on the expression of CCL20 and CCR6 in HDMECs. As shown in Fig. 2A, *FLI1* siRNA enhanced CCR6 expression on the mRNA level, while not affecting CCL20 expression. Increased expression of CCR6 by FLI1 depletion was also confirmed on the protein level by immunoblotting (Fig. 2B). Furthermore, chromatin immunoprecipitation revealed the binding of FLI1 to the *CCR6* promoters of 2 transcript variants (Transcript variant 1; NM_004367, Transcript variant 2; NM_031409) in HDMECs (Fig. 2C). These results suggest that FLI1 serves as a transcriptional repressor of the *CCR6* gene and that FLI1 deficiency at least partially contributes to CCR6 upregulation in endothelial cells.

Given that the CCL20/CCR6 axis plays a critical part in the recruitment of Th17 cells and regulatory T cells, we also evaluated the effects of cytokines produced by those cells, such as IL-17A, IL-10, and TGF-β1, on endothelial CCR6 expression. As shown in Fig. 2D, IL-17A and IL-10, but not TGF-β1, enhanced the expression of CCR6 in HDMECs. These results suggest that the recruitment of Th17 cells and regulatory T cells potentially promotes the expression of CCR6 in endothelial cells, possibly regulating the CCL20/CCR6-dependent angiogenic process related to the pathological inflammation. Considering that IL-17A is up-regulated in the skin of SSc patients [33, 34], IL-17A likely contributes to CCR6 induction in dermal microvascular endothelial cells of SSc patients, as well as FLI1 deficiency.

### *Ccr6* siRNA restores vascular hyperpermeability induced by BLM injection in mice

**Fig. 3:**
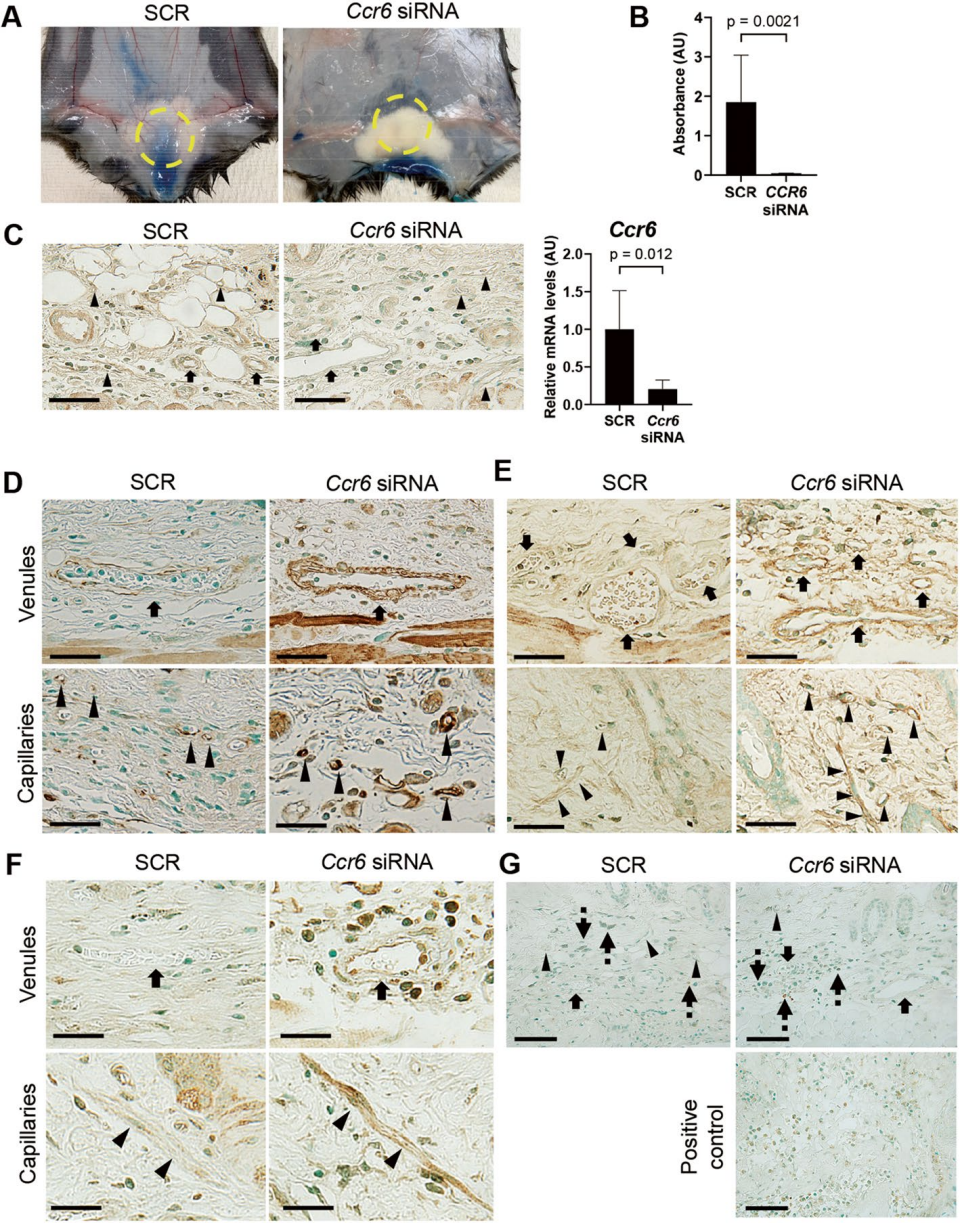
*Ccr6* gene silencing restores BLM-induced vascular hyperpermeability in mice. Wild-type mice were injected with *Ccr6* siRNA or scrambled non-silencing RNA (SCR), followed by 1-week and 4-week bleomycin (BLM) challenge. **A** The leakage of Evans blue dye was macroscopically evaluated in the skin after 1-week BLM injection. **B** In vitro transwell permeability assay showed decreased permeability in the monolayer of HDMECs treated with *CCR6* siRNA relative to the control cells treated with SCR. **C**–**F** After 4-week BLM injection, CCR6 was visualized by immunohistochemistry (left photographs of **C**). CCR6 knockdown was also confirmed by qRT-PCT with the whole skin sections (a right graph of **C**). α-smooth muscle actin (**D**), NG2 (**E**), and VE-cadherin (**F**) were visualized by immunohistochemistry. Apoptotic cells were visualized by staining cleaved caspase 3 (**G**). Skin sections of angiosarcoma were used as positive controls (a bottom panel of **G**). Unbroken arrows, arrow heads, and dashed arrows in each figure represent venules, capillaries, and inflammatory cells, respectively. Each graph indicates mean ± SEM of the indicated parameters. A scale bar is 25 μm. AU, arbitrary unit. Representative results of 6 independent experiments were shown

To further confirm if CCR6 upregulation is involved in the development of SSc vasculopathy, we evaluated the effect of *Ccr6* siRNA on the vascular aspect of BLM-treated mice, a widely recognized animal model of SSc. As a part of SSc-like vascular features induced by BLM injection, we focused on vascular permeability which can be evaluated by Evans blue dye injection [35]. In mice treated with SCR, a 1-week BLM challenge increased the permeability of vasculature in the injected skin area relative to adjacent non-injected areas (a left panel of Fig. 3A). On the other hand, Evans blue dye extravasation was remarkably attenuated after 1-week BLM injection in mice treated with *Ccr6* siRNA (a right panel of Fig. 3A). These findings were reproduced by in vitro transwell permeability assay, showing that significantly less streptavidin-HRP permeated through the monolayer of HDMECs treated with *CCR6* siRNA as compared to the control monolayer treated with SCR. (Fig. 3B). These results indicate that CCR6 is required for vascular destabilization induced by BLM-dependent inflammation.

To further confirm this finding, we employed immunostaining for α-SMA, NG2, and VE-cadherin, markers of vascular stabilization. We used the skin sections of BLM-challenged mice treated with *Ccr6* siRNA or SCR for 4 weeks, in which we confirmed CCR6 suppression at the protein level (Fig. 3C). Generally, α-SMA is highly expressed in pericytes engaged in vascular stabilization, while being marginally found in those cells promoting angiogenesis [36, 37]. As shown in Fig. 3D, the expression of α-SMA was increased in dermal small vessels (capillaries and venules) of *Ccr6* siRNA-treated mice as compared to SCR-treated ones. Another pericytes marker, NG2 proteoglycan, which enhances maturation and formation of endothelial cell junctions by acting as an auxiliary receptor that augments signaling through integrins and growth factor receptors [38], was also stained similarly (Fig. 3E). With respect to VE-cadherin, a key cell adhesion molecule regulating endothelial cell integration and vascular permeability [39], *CCR6* siRNA-treated skin exhibited stronger staining in dermal small blood vessels than SCR-treated skin (Fig. 3F). These results indicate that *Ccr6* siRNA stabilizes dermal small vessels in BLM-treated mice.

We further sought for the involvement of endothelial apoptosis in vascular permeability of BLM-treated mice because endothelial apoptosis is a part of the vascular process associated with SSc [40]. Immunostaining of cleaved caspase 3 revealed that the positive signals were totally absent in cells constituting dermal blood vessels after BLM injection under either *Ccr6* siRNA or SCR treatment, while being evident in some inflammatory cells (Fig. 3G). Thus, the increased vascular permeability was not due to apoptosis of endothelial cells. This finding was consistent with the results of earlier studies demonstrating that apoptosis is mainly observed in inflammatory cells, but not in endothelial cells and dermal fibroblasts, of BLM-treated murine skin [41].

### Decreased angiogenic activity of *CCR6* siRNA-treated HDMECs

**Fig. 4:**
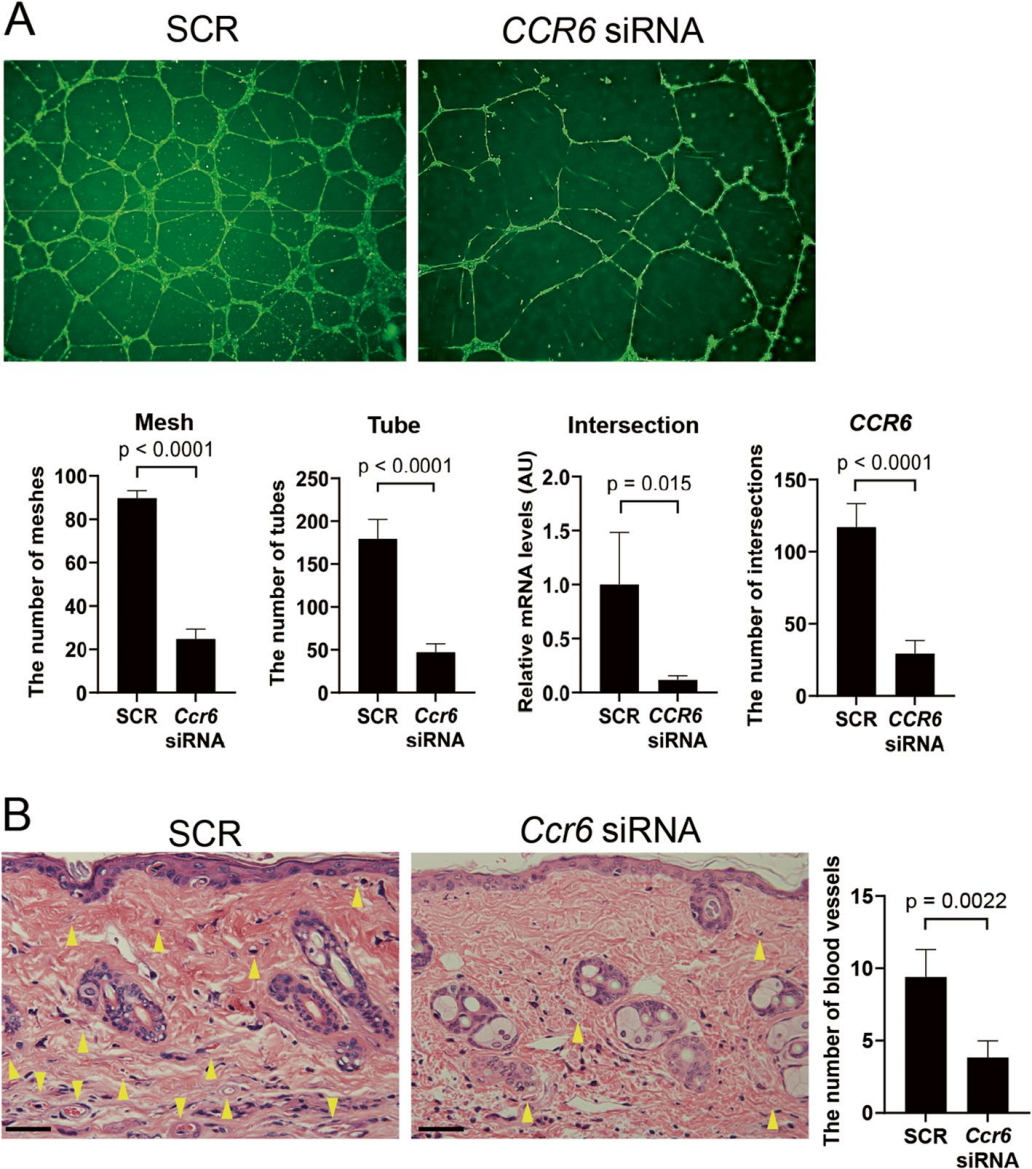
*CCR6* siRNA suppresses tubulogenic activity of HDMECs and in vivo neovascularization. **A** Tube formation assay was performed by applying HDMECs treated with *Ccr6* siRNA or scrambled non-silencing RNA (SCR) onto the Matrigel and incubating for 24 h. To eliminate the influence of proliferation, cells were treated with mitomycin C before the assay. Representative images are shown (*n* = 5 for each group). The numbers of meshes, tubes, and intersections were counted manually. *CCR6* mRNA levels were determined by qRT-PCR. **B** The number of dermal microvessels in the skin of BLM-treated mice subjected to *Ccr6* siRNA or SCR was counted manually in the specimen subjected to hematoxylin and eosin staining (*n* = 6 for each group). Each graph represents mean ± SEM of the indicated parameters

As described above, CCR6 downregulation was associated with vascular stabilization in BLM-treated mice. Generally, vascular stabilization is associated with reduced angiogenic activity, suggesting that CCR6 reduction suppresses the pro-angiogenic activity of endothelial cells. To address this issue, we employed in vitro angiogenesis assay with the Matrigel. As shown in the top panels of Fig. 4A, SCR-treated HDMECs formed favorable tube networks, whereas *CCR6* siRNA-treated HDMECs showed relatively large tube networks under the same culture condition. To objectively evaluate the activity of angiogenesis, we looked at the numbers of meshes, tubes and intersections. Of note, the numbers of meshes (91 [87–92] versus 23 [21–29.5], *p* < 0.001), tubes (180 [160.5–198] versus 45 [38.5–57], *p* < 0.001) and intersections (124 [100.5–130.5] versus 29 [20.5–38.5], *p* < 0.001) were decreased significantly in *CCR6* siRNA-treated HDMECs compared with SCR-treated HDMECs (bottom graphs of Fig. 4A). Taken together, CCR6 downregulation suppresses the pro-angiogenic activity of endothelial cells, which at least partially underlies vascular stabilization in *Ccr6* siRNA-treated BLM-injected mice. This notion was also confirmed in vivo by the decreased number of dermal small vessels in *Ccr6* siRNA-treated BLM-injected mice as compared to SCR-treated ones (Fig. 4B).

## Discussion

This study was undertaken to investigate the role of the CCL20/CCR6 axis in the development of SSc vasculopathy because serum CCL20 levels correlate with mPAP values in SSc patients [6]. Given that mPAP is positively correlated with the severity of nailfold capillary changes [42], it was postulated that the activation of the CCL20/CCR6 axis plays a part in the destabilization of dermal small vessels. Based on this idea, we evaluated the role of the CCL20/CCR6 axis in the regulation of endothelial behaviors. Our initial study with immunochemistry demonstrated the up-regulated expression of CCR6 in dermal microvascular endothelial cells of SSc patients. Further cell culture studies revealed the upregulation of CCR6 by FLI1 deficiency, a critical predisposing factor of SSc, in HDMECs. More importantly, *Ccr6* siRNA restored BLM-induced vascular hyperpermeability in mice. Taken together with evidence that the CCL20/CCR6 axis functions as a potent pro-angiogenic regulator [43], CCR6 upregulation may be involved in the development of SSc vasculopathy.

A previous study by Tao et al. [13] demonstrated the increased expression of CCL20 in dermal fibroblasts of early SSc patients relative to those cells of healthy control skin, suggesting that CCL20 produced by dermal fibroblasts contributes to Th17 infiltration into the involved skin of patients with early SSc. Supporting this previous finding, we found a significant elevation of CCL20 in dermal fibroblasts of dcSSc patients with a disease duration of < 2 years. Under the physiological condition, CCL20 is abundantly expressed by keratinocytes and endothelial cells, but its expression is relatively low in dermal fibroblasts, as shown in the current and previous studies [31]. Therefore, CCL20 upregulation seems to be a characteristic feature of SSc dermal fibroblasts. With respect to the expression levels of CCR6, our results were also consistent with the study by Tao et al. [13] in that the elevation of CCR6 was observed significantly in keratinocytes and slightly in dermal fibroblasts and infiltrated cells. In addition, our current study disclosed a significant elevation of CCR6 in dermal small vessels of SSc patients, especially in lcSSc patients, irrespective of disease duration. Taken together, these results suggest that CCL20 secreted from dermal fibroblasts plays a role in the early stage of dcSSc (disease duration of < 2 years), while CCR6 up-regulation in endothelial cells contributes to the development of SSc vasculopathy throughout the whole disease course. This notion is likely plausible because SSc-related PAH, which is linked to elevated serum CCL20 levels, is a complication frequently seen in lcSSc patients with a long disease history.

SSc vasculopathy is characterized by vascular structural changes, such as arteriolar stenosis, capillary dilation, and capillary loss [40]. The chronological capillary changes are well documented in nailfold capillaries [44]. The initial changes are capillary dilation and bleeding, reflecting vascular destabilization. These changes are followed by capillary loss, finally leading to abnormal angiogenic changes, such as ramified capillary. These vascular alterations are caused by dysregulated angiogenesis and defective vasculogenesis. FLI1 deficiency is a key disease factor regulating a broad spectrum of endothelial behaviors and vascular remodeling associated with SSc vasculopathy, including angiogenesis and vasculogenesis [45]. FLI1 deficiency suppresses the expression of CD31, VE-cadherin, S1P_1,_ and platelet-derived growth factor-B in endothelial cells, while upregulating matrix metalloproteinase-9, resulting in vascular destabilization and angiogenesis [29]. The expression of CCN1, which regulates the recruitment of circulating endothelial progenitor cells, is decreased in FLI1-deficient endothelial cells [23], at least partly contributing to defective vasculogenesis. In the current study, CCR6 was up-regulated in dermal microvascular endothelial cells of SSc-involved skin, and FLI1 deficiency increased CCR6 expression in HDMECs. Given that FLI1 bound the *CCR6* promoter in HDMECs, CCR6 is a member of molecules involved in the mechanism by which FLI1 deficiency promotes the development of SSc vasculopathy.

Chemokines are initially recognized as a family of proteins recruiting inflammatory cells to the specific tissues and organs. On the other hand, various chemokines have been shown to serve as regulators of angiogenesis associated with inflammation. For instance, CXC chemokines with glutamic acid-leucine-arginine (ELR) motif in their N terminus are potent promoters of angiogenesis, while those without ELR motif are potent inhibitors of angiogenesis [46]. Of note, the various CXC chemokines, such as CXCL4 (ELR-) [47], CXCL5 (ELR+) [26], CXCL6 (ELR+) [27], CXCL12 (ELR−) [48], CXCL13 (ELR−) [24], and CXCL14 (ELR−) [49], are thought to be associated with the development of SSc vasculopathy. With respect to CC chemokines, the broad-spectrum inhibitor of CC chemokines, 35 K, suppresses inflammation-driven angiogenesis, whereas preserving physiological ischemia-mediated angiogenesis [50]. Thus, the inhibition of chemokines may be an alternative therapeutic strategy against disease-related inflammatory vascular changes without the undesirable effects on physiological angiogenesis. Considering this point, the blockade of CCR6 is likely a potential therapeutic strategy against SSc vasculopathy. Further studies are required to clarify this point in the future.

There are several limitations in this study. The first limitation is the lack of data on the systemic inhibition of CCR6 in BLM-treated mice. This is a critical point because CCR6 is a chemoattractant receptor of Th17 cells and regulatory T cells. In this study, we used the local injection of atelocollagen mixed with *Ccr6* siRNA in the back skin of BLM-treated mice, in which CCR6 expression in circulating CD4^+^ T cells was not altered (data not shown). To elucidate the effect of systemic CCR6 inhibition, we need to investigate whether anti-CCR6 neutralizing antibody affects skin fibrosis or other vascular changes, such as PAH, using BLM-treated mice and other SSc animal models recapitulating SSc-PAH. The second limitation is that we did not assess the association of the CCR6-dependent angiogenic process and CCR6-dependent recruitment of inflammatory cells. Given that IL-17A enhanced CCR6 expression in HDMECs, Th17-skewed immune polarization may regulate the angiogenic process through CCR6 in the context of SSc pathogenesis. The third limitation is that there remains a room for discussion about the differential effects of CCR6 and CCL20 knockdown on SSc-like pathological events. We currently looked at the effect of CCR6 deletion to examine the role of the CCL20/CCR6 axis, but the targeting of CCL20 should be investigated as well in order to clarify which target is more appropriate to restore the disease process associated with CCL20/CCR6 axis in the context of SSc pathology.

## Conclusion

This is the first report demonstrating a potential contribution of CCR6 to the development of SSc vasculopathy as an inflammation-associated angiogenic factor. These findings suggest that the modification of CCR6 expression can be an effective intervention for SSc vascular symptoms. Also, the induction of CCR6 expression in FLI1-deficient endothelial cells further supports the canonical idea that FLI1 deficiency is a critical disease factor of SSc.

## Data Availability

Not applicable.

## Abbreviations

SSc: Systemic sclerosis
mPAP: Mean pulmonary arterial pressure
Th: T helper
FLI1: Friend leukemia virus integration 1
α-SMA: α-Smooth muscle actin
VE-cadherin: Vascular endothelial-cadherin
dcSSc: Diffuse cutaneous SSc
lcSSc: Limited cutaneous SSc
HDMECs: Human dermal microvascular endothelial cells
SCR: Non-silencing scrambled RNA
qRT-PCR: Quantitative reverse transcription-PCR
ChIP: Chromatin immunoprecipitation
WT: Wild-type
PAH: Pulmonary arterial hypertension
ELR: Glutamic acid-leucine-arginine
HC: Healthy controls
BLM: Bleomycin
